# Birth Outcomes of Women with Obesity Enrolled for Care at Freestanding Birth Centers in the United States

**DOI:** 10.1111/jmwh.13194

**Published:** 2020-12-30

**Authors:** Cecilia M. Jevitt, Susan Stapleton, Yanhong Deng, Xuemei Song, Kaicheng Wang, Diana R. Jolles

**Affiliations:** ^1^ University of British Columbia Vancouver British Columbia Canada; ^2^ American Association of Birth Centers Perkiomenville Pennsylvania; ^3^ Yale Center for Analytical Sciences Yale School of Public Health New Haven Connecticut; ^4^ American Association of Birth Centers Perkiomenville Pennsylvania

**Keywords:** obesity, overweight, freestanding birth centers, birth center, midwife, pregnancy, birth, perinatal obesity

## Abstract

**Introduction:**

Current US guidelines for the care of women with obesity generalize obesity‐related risks to all women regardless of overall health status and assume that birth will occur in hospitals. Perinatal outcomes for women with obesity in US freestanding birth centers need documentation.

**Methods:**

Pregnancies recorded in the American Association of Birth Centers Perinatal Data Registry were analyzed (n = 4,455) to form 2 groups of primiparous women (n = 964; 1:1 matching of women with normal body mass indices [BMIs] and women with obese BMIs [>30]), using propensity score matching to address the imbalance of potential confounders. Groups were compared on a range of outcomes. Differences between groups were evaluated using χ^2^ test for categorical variables and Student's *t* test for continuous variables. Paired *t* test and McNemar's test evaluated the differences among the matched pairs.

**Results:**

The majority of women with obese BMIs experienced uncomplicated perinatal courses and vaginal births. There were no significant differences in antenatal complications, proportion of prolonged pregnancy, prolonged first and second stage labor, rupture of membranes longer than 24 hours, postpartum hemorrhage, or newborn outcomes between women with obese BMIs and normal BMIs. Among all women with intrapartum referrals or transfers (25.3%), the primary indications were prolonged first stage or second stage (55.4%), inadequate pain relief (14.8%), client choice or psychological issue (7.0%), and meconium (5.3%). Primiparous women with obesity who started labor at a birth center had a 30.7% transfer rate and an 11.1% cesarean birth rate.

**Discussion:**

Women with obese BMIs without medical comorbidity can receive safe and effective midwifery care at freestanding birth centers while anticipating a low risk for cesarean birth. The risks of potential, obesity‐related perinatal complications should be discussed with women when choosing place of birth; however, pregnancy complicated by obesity must be viewed holistically, not simply through the lens of obesity.

## INTRODUCTION

Freestanding birth centers following American Association of Birth Centers (AABC) standards are designed for women anticipating a healthy, low‐risk pregnancy and birth.^1^ Birth centers are founded in midwifery‐led care that supports physiologic pregnancy, birth and newborn transition through health education that promotes individual responsibility and self‐care, and screening that assumes pregnancy and birth are normal until complications are evident.^1^ The most important concept in birth center organization is the separation of primary care from acute care, which requires hospitalization.^1^ Birth center philosophy and that of the midwifery model of care are closely intertwined.^1^

Obesity, currently conceptualized as white adipose tissue disease, now affects 36% of reproductive‐age women in the United States.^2^, ^3^, ^4^ Women with obese pregravid body mass indices (BMIs) are at increased risk for multiple perinatal complications including gestational diabetes, hypertensive disorders of pregnancy, prolonged pregnancy, prolonged phases of labor, postpartum hemorrhage, cesarean birth, infection, and thrombotic events.^5^, ^6^, ^7^ Newborns of women with obesity are at increased risk for macrosomia, shoulder dystocia, and stillbirth.^5‐7^ As research revealed the health risks associated with obesity, perinatal guidelines for the management of obesity in pregnancy were developed assuming that the risks warranted planning for hospital births.

American College of Obstetricians and Gynecologists (ACOG) guidelines for pregnancy complicated by obesity assume that births will occur in hospitals and that women with obesity will have intravenous access and continuous electronic fetal monitoring.^7^ Guidelines from the Royal College of Obstetricians and Gynecologists advise that class I and class II obesity (BMIs 30‐39.9) in themselves are not reasons for women to labor and birth in consultant‐led units (physician‐led hospital units) and that multiparous women who are also healthy can plan for birth in midwifery‐led units as long as clear referral paths are in place.^8^ National Institute for Health and Care Excellence guidelines from the United Kingdom identify obesity with BMI greater than 35 as the cutoff for indicating increased risk, therefore suggesting that pregnant woman with a BMI greater than 35 require continuous electronic fetal monitoring.^9^, ^10^ No guidelines outline care for women with obesity related to freestanding births centers in the United States; however, women with obesity and BMIs exceeding 40 receive care and give birth in AABC facilities, as evidenced by their inclusion in the data set used in this study.

The perinatal outcomes of women with obesity receiving antenatal care from physicians and giving birth in hospitals have been documented and analyzed for 30 years.^5^, ^6^, ^7^, ^8^ Multiple studies have reported excellent perinatal outcomes for women receiving antenatal care and giving birth in freestanding birth centers;^11^, ^12^, ^13^, ^14^, ^15^ however, the outcomes specific to women with obesity receiving care based on midwifery models and giving birth in US freestanding birth centers have not been separately examined. In this study, we compared maternal and neonatal outcomes between woman with normal weights and those with obesity admitted to US birth centers.

The goals of this study were (1) to document the pregnancy and birth outcomes of women enrolled for birth center care whose pregravid BMIs were 30 or greater compared with women enrolled for birth center care of normal BMIs and (2) to use the antepartum and intrapartum outcomes data of women with obesity to form recommendations for the care of obese women in freestanding birth centers while supporting the best perinatal outcomes.

**QUICK POINTS**

✦ The majority of women with obese body mass indices (BMIs) in this study of freestanding birth centers experienced uncomplicated perinatal courses and gave birth vaginally.
✦ There were no significant differences in the antenatal complications, proportion of prolonged pregnancy, prolonged first and second stage labor, rupture of membranes longer than 24 hours, postpartum complications including postpartum hemorrhage, or newborn outcomes between women with obese BMIs and normal BMIs.
✦ Primiparous women with obesity who started labor at a birth center had a 30.7% hospital transfer rate and an 89% vaginal birth rate.
✦ The risks of potential, obesity‐related perinatal complications should be discussed with women when choosing place of birth; however, pregnancy complicated by obesity must be viewed holistically, not simply through the lens of obesity.

## METHODS

### Study Design

Data were obtained from AABC Perinatal Data Registry.^6^ The registry is used by 59% of AABC member birth centers and 27% of all US birth centers known to AABC and includes comprehensively documented antenatal care, labor, birth, and newborn outcomes.^13^ All data from patients entering antenatal care at the birth center, regardless of planned birth location, are entered into the online repository by the primary provider or designee prospectively during the pregnancy at 4 points: initial antenatal visit; upon leaving antenatal care, either admission in labor or transfer to other provider; after birth; and at final postpartum visit. Birth centers contributing data to the Perinatal Data Registry have individual and group training workshops hosted by the AABC, use a standardized instruction manual and data dictionary with definitions of all variables, and receive periodic newsletters and e‐mails from the AABC to maintain data quality.

For this study, we extracted data from 2012 to 2015 containing the perinatal records of more than 40,000 pregnancies from 95 US birth centers. Data from planned hospital births documented in the Perinatal Data Registry were excluded. BMI data were documented in 29,076 pregnancies. Using World Health Organization definitions, BMI was calculated as weight in kilograms divided by the height (in meters squared), and normal weight was defined as a BMI of 18.5 to 24.9, overweight as a BMI of 25 to 29.9, and obesity as a BMI of 30 or greater.^4^ Prepregnancy BMIs were used in this analysis and were collected by the data registry platform based upon either patient‐reported prepregnancy weight or weight at the first antenatal visit if occurring in the first trimester of pregnancy. Use of prepregnancy BMI in the study is consistent with the National Vital Statistic System, previous literatures and guidelines for the care of women with obesity.^16^ Only primiparous women were considered in this analysis because BMI most often increases with pregnancy and the greatest risk of complications occurs with the first pregnancy and birth.^18^, ^19^ Multiparous women were also excluded, as stages of labor tend to be shorter after the first birth, confounding comparisons of length of labor.

Matching comparable baseline characteristics (Table 2) for the total subsample of women with obese BMIs using propensity score matching yielded 2 matched groups (1:1, n = 964). The final sample was small because the number of women with obesity was small. All women in the propensity match planned to give birth in a birth center.

### Outcome Variables

A range of prespecified maternal and neonatal outcomes was considered, including antenatal, intrapartum, and postpartum complications, intrapartum and postpartum maternal or neonatal transfers, and referrals to hospital as defined in the AABC Perinatal Data Registry. The definitions of many variables have changed over time or are defined differently outside of the United States; for example, postpartum hemorrhage.^13^ Definitions as used in this study can be found in Supporting Information: Appendix S1. These AABC registry definitions are harmonized with ACOG Revitalize Data definitions as well as the National Quality Forum Endorsed measures. Newborn complications viewed as sentinel events by AABC standards and the primary indications for newborn transfer to hospital as defined in the AABC Perinatal Data Registry included Apgar scores less than 7 at 5 minutes, significant respiratory issues, temperature instability, transient tachypnea, hypoglycemia, birth trauma, congenital anomalies, low birth weight, and prematurity.^19^

### Statistical Analysis

Propensity score matching, the analysis method chosen for this study, created 2 sets of participants, one exposure group (women with obese BMIs) and a nonexposed, control group (women with normal BMIs). By matching covariate characteristics, the statistical procedure attempted to approximate a random experiment and to reduce or eliminate selection bias. Primiparous women with normal BMIs and primiparous women with obese BMIs were matched with propensity scores to address the imbalance of potential confounders. The propensity score was estimated as the predicted probability of a woman being in the obese BMI group from a logistic regression model. The propensity‐score model included the categorical variables of race, insurance type, marital status, smoking status and other medical conditions, and the continuous variables age and years of education (Table 1).

**Table 1:** Comparison of Demographic Characteristics Before and After Propensity Score Matching

	Original Groups Before Propensity Match				Groups After Propensity Score Matching				
		Body Mass Index Category				Body Mass Index Category			
Characteristics	Total	Normal	Obese	*P* Value^a^	Total	Normal	Obese	Std Diff	*P* Value^b^
**Total, n (%)**	4455 (100)	3973 (89.2)	482 (10.8)		964 (100)	482 (50)	482 (50)	.0003	
**Body mass index, median (range)**	22.1 (18.5‐46.9)	21.9 (18.5‐25)	32.8 (30.0‐46.9)		27.5 (18.6‐46.9)	22.1 (18.6‐25.0)	32.8 (30.0‐46.9)		
**Age, mean (SD), y**	28.3 (4.6)	28.4 (4.5)	27.9 (4.8)	.048	28.1 (4.8)	28.2 (4.8)	27.9 (4.8)	.049	.92
**Race, n (%)**				<.001				<.0001	.98
White	3732 (83.8)	3361 (84.6)	371 (77.0)		743 (77.1)	372 (77.2)	371 (77)		
African American	171 (3.8)	130 (3.3)	41 (8.5)		83 (8.6)	42 (8.7)	41 (8.5)		
Other	552 (12.4)	482 (12.1)	70 (14.5)		138 (14.3)	68 (14.1)	70 (14.5)		
**Years of education, median (range)**	16 (0‐27)	16 (0‐27)	15.5 (0‐26)	.003	16 (0‐26)	16 (0‐20)	15.5 (0‐26)	.018	.68
**Insurance, n (%)**				<.001				.030	.77
Private insurance	2866 (64.3)	2580 (64.9)	286 (59.3)		574 (59.5)	288 (59.8)	286 (59.3)		
Public insurance	849 (19.1)	717 (18)	132 (27.4)		260 (27)	128 (26.6)	132 (27.4)		
Other	740 (16.6)	676 (17)	64 (13.3)		130 (13.5)	66 (13.7)	64 (13.3)		
**Marital status, n (%)**				.002				.014	.76
Single	944 (21.2)	816 (20.5)	128 (26.6)		253 (26.2)	125 (25.9)	128 (26.6)		
Married	3511 (78.8)	3157 (79.5)	354 (73.4)		711 (73.8)	357 (74.1)	354 (73.4)		
**History of smoking, n (%)**	173 (3.9)	140 (3.5)	33 (6.8)	<.001	65 (6.7)	32 (6.6)	33 (6.8)	<.0001	.84
**Any medical history,** ^c^ **n (%)**	523 (11.7)	435 (10.9)	88 (18.3)	<.001	177 (18.4)	89 (18.5)	88 (18.3)	.005	.88

Abbreviation: Std diff, standard difference.

^a^χ^2^ test or Student's *t* test

^b^Paired *t* test or McNemar's test

^c^Any medical history is defined in the American Association of Birth Centers Perinatal Data Registry as any history of anorexia or bulimia, asthma (requiring prescription medication or in‐patient treatment), bicornuate or septate uterus, chronic hypertension (requiring prescription medication or in‐patient treatment), chronic renal disease, client herself was born preterm (<37 weeks), depression or psychiatric illness (requiring prescription medication or in‐patient treatment), diabetes (preexisting), intimate partner violence, heart disease (class II‐IV), HIV antibody positive, periodontal disease (bleeding or receding gums, caries, lack of dental care, poor dental hygiene), seizures (requiring prescription medication or in‐patient treatment), sexual abuse or assault history, substance abuse, thrombophilia, or thyroid disease (requiring prescription medication or in‐patient treatment).

Exclusions were those listed in the AABC Perinatal Data Registry as physical and obstetric conditions that require medical referral, including anorexia or bulimia, cervical abnormality, chronic hypertension, the woman herself being born preterm, type 1 and type 2 diabetes, heart disease class II‐IV, HIV positive, infertility treatment resulting in current pregnancy, seizures requiring treatment, sexually transmitted infection in the 6 months prior to the current pregnancy, thrombophilia, and uterine abnormality. Other medical conditions included asthma and thyroid disease needing treatment and nonchronic conditions not associated with obesity such as vaginitis.

Matched pairs were formed between women in the normal BMI and obese BMI groups using a one‐to‐one optimal fixed ratio matching with caliper width of 0·1. A total of 964 women were matched with a standardized mean difference of 0.0003 in propensity score. Only women matched with propensity scores were included in the final analyses.

Categorical variables are displayed using frequency and percentage, whereas continuous variables are displayed using means (SD) or medians (range). Differences between prematched groups were evaluated using χ^2^ test for categorical variables and Student's *t* test for continuous variables. Meanwhile, paired *t* test and McNemar's test were performed to evaluate the differences in the matched groups in consideration of the correlation among the matched pairs. Standardized differences were also provided to evaluate the differences on baseline characteristics between 2 matched groups. All missing values were coded as not available and excluded from test. A *P* value of less than .05 was considered statistically significant. All statistical analyses were conducted with SAS version 9.4 (SAS Institute, Cary, NC).

### Human Subjects Protection

Written consent was obtained from all participants prior to data entry into the registry. The data set obtained contained no personal identifiers. The AABC Research Committee and Board of Directors approved this study in 2014 and the Yale University Human Subjects Committee granted an exemption for analysis of deidentified data. All data were kept in Yale encrypted computers within Yale internet firewalls, which use double password protection.

## RESULTS

### Population Characteristics

**Figure 1:**
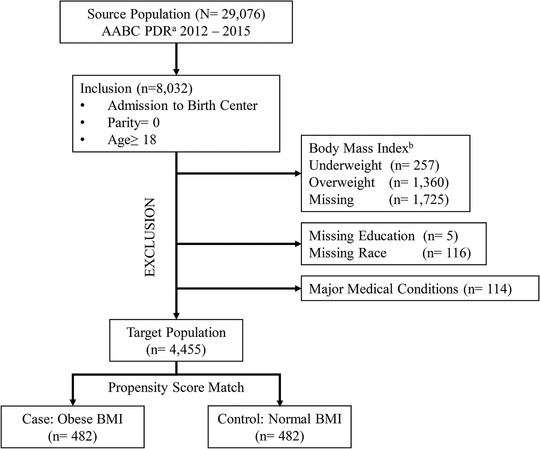
**Sample Selection from American Association of Birth Centers Perinatal Data Registry** Abbreviation: BMI, body mass index. ^a^American Association of Birth Centers Perinatal Data Registry. ^b^World Health Organization BMI categories were used: underweight, <18.5; normal weight, 18.5 to 24.9; overweight, 25 to 25.9; obese ≥30.

There were 8032 primiparous women who were 18 years older at admission to the birth center. In the end, 4455 women were left after exclusion using the criteria in Figure 1. Table 1 shows demographic and health‐related characteristics of women in the initial sample (n = 4455) and then in the propensity score‐matched sample (n = 964). In the initial sample, women with obese BMIs compared with women with normal BMIs were more likely to be identified as African American or other race (*P* < .001), single (*P* = .002), covered by public insurance (*P* < .001), and with history of smoking (*P* < .001). Moreover, women with obese BMIs were significantly more likely to have health‐related concerns prior to antenatal care enrollment than women with normal BMIs (18.3% vs 10.9%, *P* < .001). Specifically, health‐related concerns included asthma, periodontal disease, thyroid disease, urinary tract infection, and mental health concerns. After matching with propensity score, no significant differences were observed between women with normal BMIs and women with obese BMIs. The standardized difference showed that 2 groups were well‐matched and the differences were minimal (range from .005 to < .0001, Table 2).

**Table 2:** Antepartum, Intrapartum, Postpartum, and Newborn Outcomes

		Body Mass Index Category		
	Total	Normal	Obese	*P Value* ^a^
Characteristics	n = 964	n = 482	n = 482	
**Antenatal complications, n (%)**				.39
None	750 (77.8)	382 (79.2)	368 (76.3)	
Any	214 (22.2)	100 (20.7)	114 (23.7)	
**Intrapartum complications, n (%)**				.53
None	505 (52.4)	273 (56.7)	232 (48.1)	
1	298 (30.9)	140 (29.0)	158 (32.8)	
2+	161 (16.7)	69 (14.3)	92 (19.1)	
Late term pregnancy	102 (10.6)	48 (10.0)	54 (11.2)	.85
Intrapartum group B streptococcus colonization	141 (14.6)	56 (11.6)	85 (17.6)	.004
Prolonged first stage labor	150 (15.6)	65 (13.5)	85 (17.6)	.35
Prolonged second stage labor	63 (6.5)	34 (7.1)	29 (6.0)	.22
Intrapartum, rupture of membranes >24 hr	54 (5.6)	22 (4.6)	32 (6.6)	.16
**Intrapartum transfer or referral, n (%)**				.88
No	720 (74.7)	386 (80.1)	334 (69.3)	
Yes	244 (25.3)	96 (19.9)	148 (30.3)	
Intrapartum transfer to hospital, prolonged or arrest first‐ or second‐stage labor	135 (14.0)	52 (10.8)	83 (17.2)	.035
**Birth type (initial admission for labor at birth center), n (%)**				.47
Cephalic spontaneous vaginal	859 (89.6)	440 (91.7)	419 (87.5)	
Primary cesarean birth	81 (8.4)	28 (5.8)	53 (11.1)	
Instrumental (vacuum, forceps)	19 (2.0)	12 (2.5)	7 (1.5)	
**Postpartum complications, n (%)**				.31
None	869 (90.2)	431 (89.4)	438 (90.0)	
Any	95 (9.9)	51 (10.6)	44 (9.1)	
Postpartum hemorrhage	54 (5.6)	31 (6.4)	23 (4.8)	.17
Postpartum retained placenta	14 (1.5)	6 (1.2)	8 (1.7)	.27
**Postpartum transfer or referral, n (%)**				.69
No	938 (97.3)	470 (97.5)	468 (97.1)	
Yes	26 (2.7)	12 (2.5)	14 (2.9)	
**Newborn weight, mean (SD), g**	3482 (419)	3455 (400)	3508 (436)	.94
**Apgar score 1 min, n (%)**				.58
Normal (7‐10)	890 (92.4)	440 (91.3)	450 (93.4)	
Distressed	74 (7.7)	42 (8.7)	32 (6.6)	
**Apgar score 5 min, n (%)**				.34
Normal (7‐10)	953 (98.9)	477 (99.0)	476 (98.8)	
Distressed	11 (1.1)	5 (1.0)	6 (1.2)	
**Newborn transfer or referral, n (%)**				.11
No	945 (98.0)	473 (98.1)	472 (97.9)	
Yes	19 (2.0)	9 (1.9)	10 (2.1)	

^a^Paired *t* test or McNemar's test. Cases with missing data were excluded.

### Clinical Outcomes

The majority of women with obese BMIs in this study experienced uncomplicated antenatal and postpartum courses and gave birth vaginally (Table 2). The primiparous women with obese BMIs had vaginal birth rates slightly lower than those of women with normal BMIs (87.5% and 91.7%, *P* = .02). There were no significant differences in the antenatal and intrapartum complications between women with obese BMIs and normal BMIs (Table 2).

No significant differences were found in the proportions of prolonged pregnancy, prolonged first and second stage labor, rupture of membranes longer than 24 hours, or other intrapartum complications (Table 2). Prolonged first stage was the most common intrapartum complication, yet only 15.6% of all women had prolonged first stages (Table 3). Prolonged second stage was less common occurring in 6.5% of labors. The second most common complication was nonparticulate meconium (7.9%, Table 3). Hypertensive disorders occurred in 1% or less of women in both groups (Table 3). Of women accepting prophylactic antibiotics, women with obesity had higher rates of group B streptococcus (GBS) colonization (17.6% and 11.6%, *P* = .052, Table 2). There was no difference in the proportion of women with obese BMIs and those with normal BMIs who tested positive for GBS but did not receive prophylaxis before the birth (3.9% and 3.5%, *P* = .3).

**Table 3:** Intrapartum Complications

		**Body Mass Index Category**		
	**Total**	**Normal**	**Obese**	***P* Value** **b**
**Complication** **a**	**n = 964** **n (%)**	**n = 482** **n (%)**	**n = 482** **n (%)**	
Prolonged first stage	150 (15.6)	65 (13.5)	85 (17.6)	0.51
Nonparticulate meconium	76 (7.9)	31 (6.4)	45 (9.3)	0.11
Prolonged second stage	63 (6.5)	34 (7.1)	29 (6.0)	0.74
Rupture of membranes >24 hr	54 (5.6)	22 (4.6)	32 (6.6)	0.43
Indeterminate fetal heart rate pattern	50 (5.2)	24 (5.0)	26 (5.4)	0.45
Particulate meconium	38 (3.9)	19 (3.9)	19 (3.9)	1
Shoulder dystocia requiring maneuvers	24 (2.5)	12 (2.5)	12 (2.5)	0.79
Malpresentation	16 (1.7)	8 (1.7)	8 (1.7)	0.71
Gestational hypertension	11 (1.4)	5 (1.0)	6 (1.2)	0.65
Chorioamnionitis	11 (1.1)	7 (1.5)	4 (0.8)	0.096
Abnormal FHR pattern	10 (1.0)	5 (1.0)	5 (1.0)	0.48
Maternal fever	8 (0.8)	3 (0.6)	5 (1.0)	1
Placental abruption	4 (0.4)	3 (0.6)	1 (0.2)	0.15
Preeclampsia	4 (0.4)	2 (0.4)	2 (0.4)	1
Preterm labor (labor 32 to 37 wk)	4 (0.4)	1 (0.2)	3 (0.6)	0.08
Stage 3 cord avulsion	1 (0.1)	1 (0.2)	0	N/A
Cord prolapse	1 (0.1)	1 (0.2)	0	N/A
Hemolysis, elevated liver enzymes, low platelets syndrome	1 (0.1)	1 (0.2)	0	N/A
Other (any complication not listed above)	6 (0.6)	1 (0.2)	5 (1.0)	N/A

Abbreviations: FHR, fetal heart rate; N/A, not applicable.

^a^According to American Association of Birth Centers standards these conditions make birth center inappropriate and prompt hospital transfer.

^b^McNemar's test.

Overall intrapartum transfer rates were higher among women with obese BMIs than woman normal BMIs (30.7% vs 19.9%, *P* < .0001, Table 2). Prolonged first stage and inadequate pain relief were the 2 most common indications for transfers that were recorded as referrals or emergency intrapartum transfers to hospitals (Table 4). Other indications for transfers are listed in Table 4 but occurred too infrequently for significance testing. No woman from either group had very preterm labor, placenta previa, uterine hyperstimulation, severe preeclampsia, eclampsia, seizures (noneclamptic), or surgical injury during a cesarean birth. There were no maternal intensive care unit admissions and no maternal or neonatal deaths.

**Table 4:** Primary Indication for Emergency Intrapartum Transfer or Referral

		**Body Mass Index Category**	
	**Total**	**Normal**	**Obese**
Indications	**(n = 244)** **n (%)**	**n (%)** **96 (39.3)**	**n (%)** **148 (60.7)**
Prolonged or arrest: first stage	118 (48.4)	43 (44.8)	75 (50.7)
Inadequate pain relief	36 (14.8)	13 (13.5)	23 (15.5)
Prolonged or arrest: second stage	17 (7.0)	9 (9.4)	8 (5.4)
Client choice/psychological	17 (7.0)	6 (6.3)	11 (7.4)
Prolonged ROM with labor >24 hr	11 (4.5)	4 (4.2)	7 (4.7)
Gestational hypertension	11 (4.5)	4 (4.2)	7 (4.7)
Particulate meconium	11 (4.5)	4 (4.2)	7 (4.7)
Indeterminate or concerning FHR pattern	10 (4.1)	6 (6.3)	4 (2.7)
Abnormal FHR pattern	5 (2.0)	2 (2.1)	3 (2.0)
Malpresentation	3 (1.2)	2 (2.1)	1 (0.7)
Nonparticulate meconium	2 (0.8)	1 (1.0)	1 (0.7)
Prolonged latent phase	2 (0.8)	1 (1.0)	1 (0.7)
Intrapartum hemorrhage	1 (0.4)	1 (1.0)	0

Abbreviations: FHR, fetal heart rate; ROM, rupture of membranes.

The proportion of postpartum complications, including postpartum hemorrhage (6.4%, 4.8%) and retained placenta (1.2%, 1.7%), were similar in women with normal and obese BMIs (Table 2). The primary reasons for maternal postpartum transfer included laceration or episiotomy requiring repair, hypertension without evidence of preeclampsia, maternal fever, preeclampsia, postpartum hemorrhage, and retained placenta. Overall, there were no significant differences in postpartum transfer or newborn transfer to a hospital for extended care between the 2 groups. The average newborn weights (3455 vs 3506 g) and Apgar scores were similar between the 2 groups.

## DISCUSSION

Obesity in the United States is associated with poverty in a complex relationship that includes increased exposure to endocrine‐disrupting chemicals; limited ability to buy expensive nutritious foods; residence in neighborhoods without full service groceries; limited access to health care, fitness centers, or weight management programs; and neighborhoods that are unsafe for outdoor physical activity.^20^, ^21^ Consistent with US national data, in the overall data set from the AABC Perinatal Data Registry, Black or African American women had higher rates of obesity.^20^, ^21^ Although income data were not included in this study, public insurance in the United States is an indicator of low‐income status. More women with obese BMIs in the overall data set had antenatal coverage through public programs than women with normal BMIs. In this study, women with obese BMIs had higher indicators for socioeconomic risk yet had outcomes similar to women with normal BMIs.

Antenatal complications for women with obesity, including minor complications such as anemia, were low, with 77.8% having uncomplicated antenatal courses. A study of women with BMIs greater than or equal to 30 in the United Kingdom found similarly that 68% had no antenatal complications.^22^ Other studies of women with obesity receiving midwifery care focus on intrapartum and postpartum outcomes.

Women with obesity in this study had significantly higher rates of antenatal GBS colonization than women with normal BMIs (17.6%, 11.6%). The American College of Obstetricians and Gynecologists describes US rates of GBS colonization ranging from 10% to 30% varying with race and geographical location.^23^ Several studies have shown that obesity is an independent risk factor for colonization with GBS,^23^, ^24^, ^25^ with the incidence of colonization increasing significantly with each BMI unit.^24^ Colonization with GBS does not require hospitalization for births because intravenous antibiotic prophylaxis is available to women in freestanding birth centers.

Reduction of cesarean births is a national quality strategy. Women with obese BMIs receiving care based on generalized medical guidelines may be at higher risk of cesarean birth and related sequela.^26^, ^27^ In this study, the birth center model of care demonstrated a markedly lower cesarean birth rate for women with obesity compared to the medical literature and the US national cesarean rate. Women of mixed parity and BMIs admitted to birth centers in labor in one US birth center study had a 6% primary cesarean birth rate.^15^ In this current study, the cesarean birth rate for women with obese BMIs is significantly higher than women with normal BMIs (11.1% vs 5.8%, *P* = .02), yet these rates that are dramatically lower than the overall US low‐risk cesarean birth rate of 26.9% for primiparous women during the time of this study in 2013.^28^ Low risk was defined as singleton, greater than or equal to 37 weeks’ gestational age, and vertex in the 2013 analysis.^28^

Based on prior research, the cesarean birth rate for primiparous women with obesity would be expected to be higher.^7^, ^26^, ^28^ In a study of 2235 women that calculated cesarean rate by BMI, rates were 31.4% for women of normal BMIs and 40.8% for women with obese BMIs.^26^ Another study including 143,403 women found that primiparous women with normal BMIs had a 33.2% cesarean rate and women with obese BMIs had an overall rate of 47.56%.^27^ These 2 studies represented women cared for in obstetric units. In contrast, women with obese BMIs in the UK Birthplace Study had a 13.6% cesarean birth rate,^12^ and women with BMIs of at least 35 admitted to alongside birth centers in England had a 4.7% cesarean birth rate.^29^ Lower cesarean birth rates in these studies may be attributable to evidence‐based midwifery techniques including delaying admission until labor is active, using upright positions and mobility during the first stage of labor, using relaxation techniques for labor pain relief, continuous support in labor, and using water immersion during labor.^27^, ^28^, ^29^, ^30^, ^31^

Women with obese BMIs have been shown to have prolonged pregnancies, longer labors, increased use of oxytocin during labor, increased use of oxytocin doses greater than 20 mU per minute, longer oxytocin use, and increased rates of postpartum hemorrhage.^32^, ^33^, ^34^, ^35^ Altered maternal and placental hormones, particularly leptin, are implicated in the reduced cervical ripening and inhibited myometrial contractility in women with obesity.^32^, ^33^, ^34^, ^35^, ^36^ The women with obese BMIs in this propensity match did not differ from women with normal BMIs in regard to postterm pregnancies (42 weeks or greater)^19^ or length of stage 1 or stage 2 labor.

As in prior US birth center studies, the most common postpartum complications were hemorrhage and retained placenta.^13^, ^15^ Prior birth center studies did not quantify postpartum hemorrhage rates.^13^, ^15^ In this study, rates for women with obese BMIs were similar to those of women with normal BMIs (4.8%, 6.4%). Primiparous women with obese BMIs receiving midwifery care at the University of Colorado Hospital had a 16.9% postpartum hemorrhage rate using the same definition as this study.^27^ Comparison with postpartum hemorrhage rates in recent US obstetrical literature and midwifery‐led, British alongside units cannot be done, as these studies define postpartum hemorrhage as blood loss exceeding 1000 or 1500 mL following a vaginal birth. The fact that this study used a lower definition of postpartum hemorrhage (500 mL) and had only a 4.8% rate may indicate the overall health of the women with obesity or more active management of the third stage of labor.

Risk‐based care for obesity in the United States is generalized to all women with obese BMIs regardless of obesity class or comorbid conditions. This general application of guidelines overmedicalizes care. Guidelines for obesity that are not data based limit access to midwifery care for women with obesity from vulnerable circumstances, care that has been shown safe, effective, and cost‐effective.^11^, ^12^, ^13^, ^14^, ^15^ Additionally, overapplication of treatments meant to avoid obesity‐related risk may unnecessarily increase risk for iatrogenic complications such as uterine hyperstimulation during augmentation of labor with oxytocin.

With the safety of birth center care and midwifery care for women with obese BMIs demonstrated in this and other studies,^12^, ^29^, ^30^, ^32^ attention must turn to the potential individual and health systems savings that could be realized if more women with obesity chose birth center care. Birth center care reduces costs not only through reducing adverse outcomes but by reduced length of stay and lower supply use. The US federal Strong Start for Mothers and Newborns initiative analyzed outcomes from alternative maternity care models including freestanding birth centers, finding that women who received prenatal care in birth centers had lower rates of preterm and low birth weight newborns and lower rates of cesarean births.^37^ Total average expenditures for women receiving care in the Strong Start group were $2010 lower than costs for women in comparison groups.^37^ With more than one‐third of reproductive‐aged women having obese BMIs, a shift to greater use of birth centers could produce substantial savings.

### Strengths and Limitations

This novel study provides insight from midwifery about women with obesity receiving care and giving birth at freestanding birth centers in the United States. The demographic characteristics of women in this study mirror findings in US obesity research, increasing generalizability.^3^, ^20^ To increase comparability with most perinatal obesity research that used prepregnancy BMI to calculate obesity‐associated risk, only prepregnancy BMI was used to define the groups in this study. It is unknown how the cases where BMI were not recorded compare with those where BMI was recorded. The comparability of birth centers that contributed data to those that did not is unknown. Excess gestational weight gain was not considered although this is known to increase maternal and fetal risk for adverse perinatal outcomes.^7^ Additionally, generalizability of these outcomes is limited to women receiving care in the midwifery and birth center models. Further research is needed on the pregnancy and birth outcomes of women with obese BMIs receiving midwifery care in general populations.

## CONCLUSION

This study adds evidence on the safety and benefits of midwifery‐provided antenatal care at freestanding births centers for women with obese BMIs, as well as intrapartum and postpartum and birth care for obese women without medical comorbidity.^11^, ^12^, ^15^, ^27^ The risks of intrapartum and postpartum complications and hospital referral or emergency transfer along with cesarean birth risk should be carefully evaluated and discussed with women with obese BMIs when choosing place of birth. Pregnancy complicated by obesity must be viewed holistically, with overall health considered in addition to BMI measurement. Additional research analyzing perinatal outcomes for otherwise healthy women with different classes of obesity will allow for updated perinatal care guidelines to reflect the breadth of potential outcomes for women with obesity.

## CONFLICT OF INTEREST

The authors have no conflicts of interest disclose.

## Supporting information


**Appendix S1**. Variables As Defined in the American Association of Birth Centers Perinatal Data Registry

## References

[1] American Association of Birth Centers . Definition of a Birth Center. Accessed July 6, 2020. http://www.birthcenters.org/?page=bce_what_is_a_bc

[2] Coelho M , Oliveira T , Fernandes R . Biochemistry of adipose tissue: an endocrine organ. Arch Med Sci. 2013;9(2):191‐200. 23671428 10.5114/aoms.2013.33181 PMC3648822

[3] Hales CM , Carroll MD , Fryar CD , Ogden CL . *Prevalence of Obesity Among Adults and Youth: United States, 2015‐2016: NCHS Data Brief, no 288*. National Center for Health Statistics; 2017. 29155689

[4] World Health Organization . Obesity and overweight. 2019. Accessed July 6, 2020. https://www.who.int/news-room/fact-sheets/detail/obesity-and-overweight

[5] Catalano PM , Shankar K. Obesity and pregnancy: mechanisms of short term and long term adverse consequences for mother and child. BMJ. 2017. 8;356:j1. 10.1136/bmj.j1 PMC6888512 28179267

[6] Santangeli L , Sattar N , Huda S . Impact of maternal obesity on perinatal and childhood outcomes. Best Pract Res Clin Obstet Gynaecol. 2015. 29(3):438‐448. 25497183 10.1016/j.bpobgyn.2014.10.009

[7] American College of Obstetricians and Gynecologists . ACOG committee opinion #549: obesity in pregnancy. Obstet Gynecol. 2013;121(1)213‐217. 23262963 10.1097/01.aog.0000425667.10377.60

[8] Denison FC , Aedla NR , Keag O , et al; Royal College of Obstetricians and Gynaecologists. Care of women with obesity in pregnancy. Green‐top guideline no. 72. BJOG 2018;126(3):e62‐e106. 30465332 10.1111/1471-0528.15386

[9] National Institute for Health and Care Excellence . Intrapartum care for women with existing medical conditions or obstetrics complications and their babies‐obesity. 2019. Accessed July 6, 2020. https://www.nice.org.uk/guidance/ng121/chapter/Recommendations#obesity 31194308

[10] National Institute for Health and Care Excellence . Weight management before, during and after pregnancy: public health guideline [PH27]. 2010. Updated 2017. Accessed July 6, 2020. https://www.nice.org.uk/guidance/ph27/resources/surveillance-report-2017-weight-management-before-during-and-after-pregnancy-2010-nice-guideline-ph27-4424111104/chapter/Surveillance-decision

[11] Rooks J , Weatherby N , Ernst E , Stapleton S , Rosen D , Rosenfield A . Outcomes of care in birth centers: the national birth center study. N Engl J Med. 1989;321(26):1804‐1811. 2687692 10.1056/NEJM198912283212606

[12] Hollowell J , Li Y , Bunch K , Brocklehurst P . A comparison of intrapartum interventions and adverse outcomes by parity in planned freestanding midwifery unit and alongside midwifery unit births: secondary analysis of “low risk” births in the birthplace in England cohort. BMC Pregnancy Childbirth. 2017;17(1):95. 28320352 10.1186/s12884-017-1271-2 PMC5359981

[13] Alliman J , Phillippi J. Maternal outcomes in birth centers: an integrative review. J Midwifery Womens Health. 2016;61(1):21‐51. 26773853 10.1111/jmwh.12356

[14] Phillippi J , Danhausen D , Alliman J , Phillippi R . Neonatal outcomes in the birth center setting: a systematic review. J Midwifery Womens Health. 2018;63(1):68‐89. 29419926 10.1111/jmwh.12701

[15] Stapleton S , Osborne C , Illuzzi J . Outcomes of care in birth centers: demonstration of a durable model. J Midwifery Womens Health. 2013;58(1):3‐14. 23363029 10.1111/jmwh.12003

[16] Institute of Medicine . Weight Gain During Pregnancy: Reexamining the Guidelines. The National Academies Press; 2009. 20669500

[17] Hill B , Bergmeier H , McPhie S , et al. Is parity a risk factor for excessive weight gain during pregnancy and postpartum weight retention? A systematic review and meta‐analysis. Obes Rev. 2017;18(7):755‐764. 28512991 10.1111/obr.12538

[18] Hamilton BE , Martin JA , Osterman MJK , Driscoll AK , Rossen LM . Births: provisional data for 2016. Vital Statistics Rapid Release. no 2. National Center for Health Statistics. 2017. Accessed July 6, 2020. Available from: https://www.cdc.gov/nchs/data/vsrr/report002.pdf

[19] American Association of Birth Centers . Perinatal data registry. Accessed July 6, 2020. http://www.birthcenters.org/PDR

[20] Cassidy‐Bushrow AE , Peters RM , Burmeister C , Bielak LF , Johnson DA . Neighborhood‐level poverty at menarche and prepregnancy obesity in African‐American women. J Pregnancy. 2016; 2016:4769121. 27418977 10.1155/2016/4769121 PMC4932170

[21] Jevitt CM , Obesity and socioeconomic disparities: rethinking causes and perinatal care. J Perinat Neonatal Nurs. 2019;33(2):126‐135. 31021937 10.1097/JPN.0000000000000400

[22] Vieira M , White SL , Patel N , et al. Prediction of uncomplicated pregnancies in obese women: a prospective multicenter study. BMC Medicine. 2017;15(1):194. 29096631 10.1186/s12916-017-0956-8 PMC5669007

[23] Prevention of group B streptococcal early‐onset disease in newborns. ACOG committee opinion no. 797. Obstet Gynecol. 2020;135(2):e51‐72. 31977795 10.1097/AOG.0000000000003668

[24] Alvareaza MD , Subramanium A , Tang Y , Edwards RK . Obesity as an independent risk factor for group B streptococcal colonization. J Matern Fetal Neonatal Med. 2017;30(23):2876‐2879. 27997266 10.1080/14767058.2016.1265937

[25] Kleweis SM , Cahill AG , Odibo AO , Tuuli MG . Maternal obesity and rectovaginal group B streptococcus colonization at term. Infec Dis Obstet Gynecol. 2015;2015:586767. 26300620 10.1155/2015/586767 PMC4537723

[26] Kawakita T , Reddy UM , Landy HJ , Igbal SN , Huang CC , Grantz KL . Indications for primary cesarean delivery relative to body mass index. Am J Obstet Gynecol. 2016:215(4): 515.e1‐9. 27210064 10.1016/j.ajog.2016.05.023 PMC5045770

[27] Carlson N , Corwin E , Lowe N . Labor intervention and outcomes in women who are nulliparous and obese: comparison to nurse‐midwife to obstetrician intrapartum care. J Midwifery Womens Health. 2017;62(1):29‐39. 28099786 10.1111/jmwh.12579 PMC5726520

[28] Osterman MJK , Martin JA . Trends in low‐risk cesarean delivery in the United States, 1990‐2013. Natl Vital Stat Rep. 2014;63(6):1‐16. 25383560

[29] Rowe R , Knight M , Kurinczuk JJ ; UK Midwifery Study System (UKMidSS). Outcomes for women with BMI>35kg/m**^2^** admitted for labour care to alongside midwifery units in the UK: a national prospective cohort study using the UK Midwifery Study System (UKMidSS). PLoS One. 2018;13(2):e0208041. 30513088 10.1371/journal.pone.0208041 PMC6279017

[30] Refrew M , McFadden A , Bastos MH , et al. Midwifery and quality care: findings from a new evidence informed framework for maternal and newborn care. Lancet. 2014;384(9948):1129‐1145. 24965816 10.1016/S0140-6736(14)60789-3

[31] Carlson NS , Breman R , Neal JL , Phillippi J . Preventing cesarean birth in women with obesity: influence of unit‐level midwifery presence on use of cesarean among women in the Consortium on Safe Labor Data Set. J Midwifery Womens Health. 2019;65(1):22‐32. 31464045 10.1111/jmwh.13022 PMC7021572

[32] Hajagos‐Tóth J , Ducza E , Samavathi R , Vari SG , Gaspar R . Obesity in pregnancy: a novel concept on the roles of adipokines in uterine contractility. Croat Med J. 2017;58(2):96‐104. 28409493 10.3325/cmj.2017.58.96 PMC5410735

[33] Azais H , Leroy A. , Chesquiere L , Deruelle P , Hanssens S. Effects of adipokines and obesity on uterine contractility. Cytokine Growth Factor Rev. 2017; 34:59‐66. 28389056 10.1016/j.cytogfr.2017.01.001

[34] Maeder A , Vonderheid SC , Park CG , et al. Titration of intravenous oxytocin infusion for postdates induction of labor across body mass index group. J Obstet Gynecol Neonatal Nurs. 2017; 46(4):494‐507. 10.1016/j.jogn.2017.02.006 28528810

[35] Ellis JA , Brown CM , Barger B , Carlson NS . Influence of maternal obesity on labor induction: a systematic review and meta‐analysis. J Midwifery Womens Health. 2019;64(1):55‐67. 30648804 10.1111/jmwh.12935 PMC6758543

[36] Carlson NS , Hernandez T , Hurt JK . Parturition dysfunction in obesity: time to target the pathobiology. Reprod Biol Endocrinol. 2015; 13:135. 26684329 10.1186/s12958-015-0129-6 PMC4683915

[37] Dubay L , Hill I , Garrett B , Blavin F . Improving birth outcomes and lowering costs for women on medicaid: impacts of ‘strong start for mothers and newborns’. Health Aff. 2020;39(6):1042‐1050. 10.1377/hlthaff.2019.01042 32479222

