# Supplementary material for: Birth Outcomes of Women with Obesity Enrolled for Care at Freestanding Birth Centers in the United States
Source: J Midwifery Womens Health. 2020 Dec 30;66(1):14–23. doi: 10.1111/jmwh.13194 (PMC7986149; doi:10.1111/jmwh.13194)
Supplement: Supplementary file 1 — Appendix S1. Variables As Defined in the American Association of Birth Centers Perinatal Data Registry [file JMWH-66-14-s001.docx]

**Supporting Information: Appendix S1. Variables As Defined in the American Association of Birth Centers Perinatal Data Registry**

| **Variable** | **Definition** |
| --- | --- |
| Abnormal Fetal Heart Rate Pattern | Equivalent to National Institute of Child Health and Human Development Category III. Fetal heart rate pattern on intermittent auscultation that warrants emergent transfer to hospital for continuous electronic fetal monitoring and intervention, such as prolonged bradycardia, severe variables, late decelerations |
| Major Medical Conditions | Any prepregnancy medical condition that would preclude birth center care including anorexia/bulimia, cervical abnormality, chronic hypertension, the woman herself being born preterm, type 1 and type 2 diabetes mellitus, heart failure class II-IV, HIV positive, infertility treatment resulting in current pregnancy, seizures requiring treatment, sexually transmitted infection in the 6 months prior to the current pregnancy, thrombophilia and uterine abnormality |
| Other Medical History | Asthma, Thyroid Disease, Periodontal Disease, and other relevant medical history defined by Perinatal Data Registry |
| Client choice/Psychological | There was no medical or obstetrical indication for hospital delivery, patient elected to go to the hospital or provider decided that patient needed hospital birth for social or psychological reasons that arose during labor |
| Eclampsia | Seizures in individuals meeting criteria for pre-eclampsia |
| Gestational hypertension | Systolic blood pressure >140 mm Hg and diastolic blood pressure > 90mmHg, occurring after 20 weeks gestation without proteinuria |
| HELLP Syndrome | Hemolysis, elevated liver enzymes, and low platelet count, with our without criteria for pre-eclampsia |
| Inadequate Pain Relief | Client requests medical pain relief not available at birth center (nitrous oxide not used in this data set) |
| Indeterminate or Concerning Fetal Heart Rate (FHR) Pattern | Equivalent to National Institute for Child Health and Human Development Category II. Fetal heart rate pattern is concerning enough to warrant transfer to hospital for continuous electronic fetal monitoring. |
| Intrapartum bleeding | Bleeding greater than expected for bloody show |
| Macrosomia | An infant weighing > 4500g (9.9#) at 37 weeks or greater |
| Malpresentation | Breech, face, brow, compound or transverse lie. Not malposition, i.e. occiput posterior |
| Maternal Fever | Maternal fever > 100.4 or 38C |
| Neonatal Death | Infant born alive who died within the first 28 days of life |
| Non-particulate Meconium | Meconium stained amniotic fluid without solid particles-thin meconium |
| Other Medical Conditions | Non-chronic conditions, not associated with obesity that are within the scope of midwifery care including vaginitis, cystitis, hyperemesis |
| Particulate Meconium | Meconium stained amniotic fluid with solid particles-thick meconium |
| Postpartum Hemorrhage | Estimate blood loss for vaginal birth >500 mL or > 1000 mL for cesarean section |
| Preeclampsia | Systolic blood pressure > 140mm Hg and diastolic blood pressure >90 mm Hg after 20 weeks gestation AND proteinuria (0.3 grams protein or more in a 24-hour specimen) |
| Preterm Labor | Active labor prior to 37 weeks gestation |
| Prolonged Latent Phase | Painful uterine contractions with little or no cervical change and prior to the active phase of labor |
| Prolonged/Arrest of Labor 1st Stage | Slower than expected labor progress or patient in active labor and has had cervical change, but then has no further progress for at least two hours |
| Prolonged/Arrest of Labor 2nd Stage | Slower than expected descent or no descent after 2 hours for primigravida and 1-hour for multigravida without epidural or after 3-hours for primigravida with epidural |
| Retained Placenta | Placenta required manual removal or other out-of-the-ordinary 3^rd^ stage interventions, regardless of the length of the 3^rd^ stage |
| Severe pre-eclampsia | Meets criteria for pre-eclampsia with one or more of the following: systolic blood pressure >160 mm Hg or diastolic BP > 110 mm Hg on 2 occasions at least 6 hours apart, proteinuria > 5 grams in 24 hour specimen or > 3+ on dipstick from 2 random specimens at least 4 hours apart, pulmonary edema, oliguria (< 400 mL in 24 hours), persistent headaches, epigastric pain or abnormal liver function tests, oligohydramnios or intrauterine growth restriction |
| Shoulder Dystocia | More than usual measure required to accomplish birth of shoulders once head has delivered. |
| Uterine Hyperstimulation | Greater than 5 uterine contractions in 10 minutes for two or more consecutive 10 minute periods and with late fetal heart rate decelerations or fetal tachycardia greater than 160 beats per minute or other concerning fetal heart rate changes |
